# 4-(3,4-Dimethyl-5-phenyl-1,3-oxazolidin-2-yl)-2-methoxy­phenol

**DOI:** 10.1107/S1600536810018891

**Published:** 2010-05-26

**Authors:** Mohd Razip Asaruddin, Habibah A Wahab, Nornisah Mohamed, Jia Hao Goh, Hoong-Kun Fun

**Affiliations:** a Pharmaceutical Design and Simulation Laboratory, School of Pharmaceutical Sciences, Universiti Sains Malaysia, 11800 USM, Penang, Malaysia; b Institute of Pharmaceutical and Neutraceuticals, Malaysia Ministry of Science and Technology and Innovation, Science Complex, 11900, Penang, Malaysia; c X-ray Crystallography Unit, School of Physics, Universiti Sains Malaysia, 11800 USM, Penang, Malaysia

## Abstract

In the title compound, C_18_H_21_NO_3_, the oxazolidine ring adopts an envelope conformation with the N atom at the flap position. The two benzene rings make dihedral angles of 74.27 (14) and 73.26 (15)° with the mean plane through the oxazolidine ring. In the crystal structure, O—H⋯O and C—H⋯O hydrogen bonds connect adjacent mol­ecules into chains along [010] incorporating *R*_2_^2^(8) loops and further stabilization is provided by weak inter­molecular C—H⋯π inter­actions.

## Related literature

For general background to and applications of the title oxazolidine compound, see: Fitzgerald *et al.* (2005 ▶); Kamat *et al.* (2000 ▶); Kumar *et al.* (2004 ▶); Walton *et al.* (2003 ▶). For graph-set descriptions of hydrogen-bond ring motifs, see: Bernstein *et al.* (1995 ▶). For a related structure, see: Duffy *et al.* (2004 ▶). For bond-length data, see: Allen *et al.* (1987 ▶). For the stability of the temperature controller used for the data collection, see: Cosier & Glazer (1986 ▶).
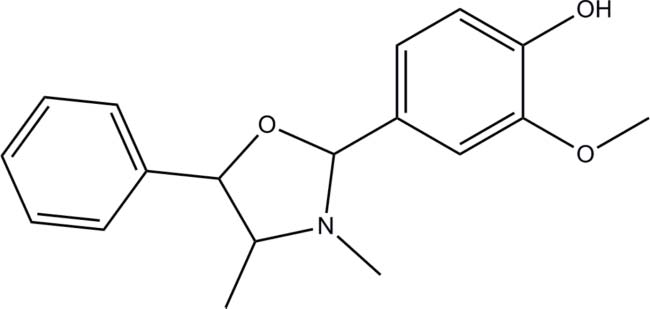


## Experimental

### 

#### Crystal data

C_18_H_21_NO_3_
*M* *_r_* = 299.36
Orthorhombic, 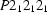

*a* = 7.8893 (6) Å
*b* = 11.7697 (9) Å
*c* = 17.4392 (13) Å
*V* = 1619.3 (2) Å^3^
*Z* = 4
Mo *K*α radiation
μ = 0.08 mm^−1^
*T* = 120 K
0.31 × 0.15 × 0.15 mm

#### Data collection

Bruker SMART APEXII CCD diffractometer
Absorption correction: multi-scan (*SADABS*; Bruker, 2009 ▶) *T*_min_ = 0.975, *T*_max_ = 0.987
9140 measured reflections
2131 independent reflections
1622 reflections with *I* > 2σ(*I*)
*R*_int_ = 0.067

#### Refinement

*R*[*F* ^2^ > 2σ(*F* ^2^)] = 0.043
*wR*(*F* ^2^) = 0.096
*S* = 1.07
2131 reflections
206 parameters
H atoms treated by a mixture of independent and constrained refinement
Δρ_max_ = 0.19 e Å^−3^
Δρ_min_ = −0.21 e Å^−3^

### 

Data collection: *APEX2* (Bruker, 2009 ▶); cell refinement: *SAINT* (Bruker, 2009 ▶); data reduction: *SAINT*; program(s) used to solve structure: *SHELXTL* (Sheldrick, 2008 ▶); program(s) used to refine structure: *SHELXTL*; molecular graphics: *SHELXTL*; software used to prepare material for publication: *SHELXTL* and *PLATON* (Spek, 2009 ▶).

## Supplementary Material

Crystal structure: contains datablocks global, I. DOI: 10.1107/S1600536810018891/hb5455sup1.cif

Structure factors: contains datablocks I. DOI: 10.1107/S1600536810018891/hb5455Isup2.hkl

Additional supplementary materials:  crystallographic information; 3D view; checkCIF report

## Figures and Tables

**Table 1:** Hydrogen-bond geometry (Å, °) *Cg*1 is the centroid of the C10–C15 phenyl ring.

*D*—H⋯*A*	*D*—H	H⋯*A*	*D*⋯*A*	*D*—H⋯*A*
O1—H1*O*1⋯O3^i^	0.92 (4)	2.08 (3)	2.909 (3)	148 (3)
C5—H5*A*⋯O1^ii^	0.93	2.42	3.244 (3)	148
C16—H16*A*⋯*Cg*1^iii^	0.96	2.91	3.628 (3)	133

Symmetry codes: (i) 
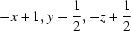
; (ii) 
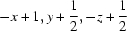
; (iii) 
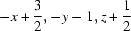
.

**Fig. 1.**
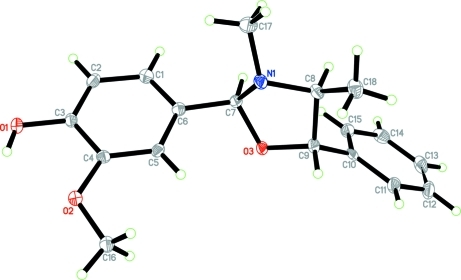
The molecular structure of (I) showing 30% probability displacement ellipsoids for non-H atoms.

**Fig. 2.**
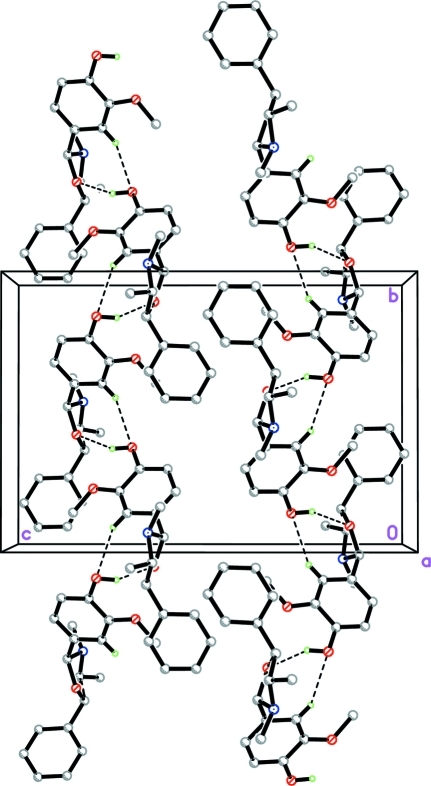
The crystal structure of (I), viewed along the a axis, showing one-dimensional chains along the [010] direction. Hydrogen atoms not involved in intermolecular interactions (dashed lines) have been omitted for clarity.

## supplementary crystallographic information

### Comment

4-Hydroxy-3-methoxybenzyldehyde has been used as a chemical intermediate in the production of pharmaceuticals and other chemicals. This compound is a widely used flavouring compound in food and personal products. The synthesis of new design chemical entity is part of the aim to produce pharmaceutical substances because the compound in the literature shown remarkable biological activities, such as anti-oxidant and anti-microbial properties (Fitzgerald *et al.*, 2005; Kamat *et al.*, 2000; Walton *et al.*, 2003; Kumar *et al.*, 2004). In this paper we report the full account of the structural data of the title oxazolidine compound, (I).

The title oxazolidine compound contains two aromatic phenyl rings bridged by an oxazolidine ring (Fig. 1). The oxazolidine ring with atom sequence C7/N1/C8/C9/O3 adopts an envolope conformation, with puckering parameters of Q = 0.421 (3) Å and φ = 73.7 (3)°. The N1 atom is at the envelope flap position and it deviates from the least-square plane through the remaining four atoms by 0.634 (2) Å. The mean plane through the oxazolidine ring inclines at dihedral angles of 74.27 (14) and 73.26 (15)°, respectively, with the C1-C6 and C10-C15 phenyl rings. The bond lengths (Allen *et al.*, 1987) and angles are within normal ranges and consistent to a closely related oxazolidine structure (Duffy *et al.*, 2004).

In the crystal structure (Fig. 2), adjacent molecules are connected by intermolecular O1—H1O1···O3 and C5—H5A···O1 hydrogen bonds (Table 1) into one-dimensional chains along the [010] direction incorporating *R*^2^_2_(8) hydrogen bond ring motifs (Bernstein *et al.*, 1995). The crystal structure is further stabilized by weak intermolecular C16—H16A···*Cg*1 interactions (Table 1) involving the centroid of the C10-C15 phenyl ring.

### Experimental

An anhydrous methanol solution of 4-hydroxy-3-methoxy-benzyldehyde (1.52 g, 10 mmol) was added to an anhydrous methanol solution of 2-methylamino-1-phenylpropan-1-ol (1.65 g, 10 mmol) and the reaction mixture was refluxed and stirred at 350 K for 8 h. The product was isolated and recrystallized from methanol and dried *in vacuo* to give colourless blocks of (I) in 80 % yield, which were washed three times with ethyl acetate and dried in a vacuum desiccator using CaCl_2_.

### Refinement

Atom H1O1 was located from difference Fourier map and allowed to refine freely [O1—H1O1 = 0.92 (3) Å]. All other H atoms were placed in their calculated positions, with C—H = 0.93 – 0.98 Å, and refined using a riding model, with *U*_iso_ = 1.2 or 1.5 *U*_eq_(C). A rotating group model was used for the methyl groups.

### Crystal data

**Table:**

C_18_H_21_NO_3_	*F*(000) = 640
*M**_r_* = 299.36	*D*_x_ = 1.228 Mg m^−^^3^
Orthorhombic, *P*2_1_2_1_2_1_	Mo *K*α radiation, λ = 0.71073 Å
Hall symbol: P 2ac 2ab	Cell parameters from 2249 reflections
*a* = 7.8893 (6) Å	θ = 2.3–30.0°
*b* = 11.7697 (9) Å	µ = 0.08 mm^−^^1^
*c* = 17.4392 (13) Å	*T* = 120 K
*V* = 1619.3 (2) Å^3^	Block, colourless
*Z* = 4	0.31 × 0.15 × 0.15 mm

### Data collection

**Table:**

Bruker SMART APEXII CCD diffractometer	2131 independent reflections
Radiation source: fine-focus sealed tube	1622 reflections with *I* > 2σ(*I*)
graphite	*R*_int_ = 0.067
φ and ω scans	θ_max_ = 27.5°, θ_min_ = 2.3°
Absorption correction: multi-scan (*SADABS*; Bruker, 2009)	*h* = −10→10
*T*_min_ = 0.975, *T*_max_ = 0.987	*k* = −15→12
9140 measured reflections	*l* = −22→20

### Refinement

**Table:**

Refinement on *F*^2^	Primary atom site location: structure-invariant direct methods
Least-squares matrix: full	Secondary atom site location: difference Fourier map
*R*[*F*^2^ > 2σ(*F*^2^)] = 0.043	Hydrogen site location: inferred from neighbouring sites
*wR*(*F*^2^) = 0.096	H atoms treated by a mixture of independent and constrained refinement
*S* = 1.07	*w* = 1/[σ^2^(*F*_o_^2^) + (0.0389*P*)^2^ + 0.1539*P*] where *P* = (*F*_o_^2^ + 2*F*_c_^2^)/3
2131 reflections	(Δ/σ)_max_ < 0.001
206 parameters	Δρ_max_ = 0.19 e Å^−^^3^
0 restraints	Δρ_min_ = −0.21 e Å^−^^3^

### Special details

**Table:**

Experimental. The crystal was placed in the cold stream of an Oxford Cryosystems Cobra open-flow nitrogen cryostat (Cosier & Glazer, 1986) operating at 120.0 (1)K.
Geometry. All esds (except the esd in the dihedral angle between two l.s. planes) are estimated using the full covariance matrix. The cell esds are taken into account individually in the estimation of esds in distances, angles and torsion angles; correlations between esds in cell parameters are only used when they are defined by crystal symmetry. An approximate (isotropic) treatment of cell esds is used for estimating esds involving l.s. planes.
Refinement. Refinement of F^2^ against ALL reflections. The weighted R-factor wR and goodness of fit S are based on F^2^, conventional R-factors R are based on F, with F set to zero for negative F^2^. The threshold expression of F^2^ > 2sigma(F^2^) is used only for calculating R-factors(gt) etc. and is not relevant to the choice of reflections for refinement. R-factors based on F^2^ are statistically about twice as large as those based on F, and R- factors based on ALL data will be even larger.

### Fractional atomic coordinates and isotropic or equivalent isotropic displacement parameters (Å^2^)

**Table:**

	*x*	*y*	*z*	*U*_iso_*/*U*_eq_	
O1	0.6439 (2)	−0.37357 (17)	0.20663 (13)	0.0265 (5)	
O2	0.5613 (2)	−0.21380 (16)	0.30639 (11)	0.0268 (5)	
O3	0.2181 (2)	0.07593 (15)	0.14249 (11)	0.0212 (4)	
N1	0.0098 (3)	−0.05656 (19)	0.15472 (13)	0.0233 (5)	
C1	0.3559 (4)	−0.2052 (2)	0.08866 (16)	0.0253 (7)	
H1A	0.3145	−0.2043	0.0387	0.030*	
C2	0.4683 (3)	−0.2903 (2)	0.11104 (17)	0.0255 (6)	
H2A	0.4999	−0.3467	0.0765	0.031*	
C3	0.5324 (3)	−0.2906 (2)	0.18443 (17)	0.0215 (6)	
C4	0.4854 (3)	−0.2053 (2)	0.23596 (16)	0.0206 (6)	
C5	0.3704 (3)	−0.1229 (2)	0.21429 (16)	0.0207 (6)	
H5A	0.3363	−0.0678	0.2493	0.025*	
C6	0.3054 (3)	−0.1223 (2)	0.13965 (16)	0.0210 (6)	
C7	0.1731 (3)	−0.0363 (2)	0.11753 (16)	0.0203 (6)	
H7A	0.1583	−0.0366	0.0617	0.024*	
C8	−0.0791 (3)	0.0517 (2)	0.14323 (17)	0.0234 (6)	
H8A	−0.1136	0.0585	0.0894	0.028*	
C9	0.0619 (3)	0.1373 (2)	0.16032 (15)	0.0217 (6)	
H9A	0.0606	0.1547	0.2153	0.026*	
C10	0.0490 (3)	0.2465 (2)	0.11626 (16)	0.0204 (6)	
C11	−0.0056 (4)	0.3445 (2)	0.15220 (18)	0.0284 (7)	
H11A	−0.0288	0.3435	0.2045	0.034*	
C12	−0.0263 (4)	0.4449 (3)	0.1110 (2)	0.0365 (8)	
H12A	−0.0623	0.5105	0.1358	0.044*	
C13	0.0064 (4)	0.4468 (3)	0.0336 (2)	0.0355 (8)	
H13A	−0.0090	0.5134	0.0058	0.043*	
C14	0.0625 (4)	0.3493 (3)	−0.00306 (18)	0.0328 (7)	
H14A	0.0852	0.3504	−0.0554	0.039*	
C15	0.0844 (4)	0.2504 (3)	0.03850 (17)	0.0264 (7)	
H15A	0.1236	0.1854	0.0139	0.032*	
C16	0.5385 (4)	−0.1212 (2)	0.35869 (16)	0.0275 (6)	
H16A	0.6074	−0.1333	0.4033	0.041*	
H16B	0.4215	−0.1169	0.3736	0.041*	
H16C	0.5713	−0.0516	0.3342	0.041*	
C17	−0.0798 (4)	−0.1538 (3)	0.12300 (19)	0.0343 (8)	
H17A	−0.0103	−0.2204	0.1276	0.051*	
H17C	−0.1044	−0.1402	0.0699	0.051*	
H17D	−0.1838	−0.1651	0.1506	0.051*	
C18	−0.2325 (3)	0.0658 (3)	0.19449 (18)	0.0320 (7)	
H18A	−0.3176	0.0114	0.1803	0.048*	
H18D	−0.2771	0.1412	0.1888	0.048*	
H18B	−0.2001	0.0538	0.2469	0.048*	
H1O1	0.658 (4)	−0.368 (3)	0.259 (2)	0.048 (10)*	

### Atomic displacement parameters (Å^2^)

**Table:**

	*U*^11^	*U*^22^	*U*^33^	*U*^12^	*U*^13^	*U*^23^
O1	0.0250 (10)	0.0216 (11)	0.0331 (12)	0.0061 (8)	−0.0054 (9)	−0.0006 (10)
O2	0.0282 (10)	0.0223 (10)	0.0299 (11)	0.0039 (8)	−0.0080 (9)	−0.0025 (9)
O3	0.0145 (8)	0.0178 (9)	0.0314 (11)	0.0008 (7)	−0.0010 (8)	−0.0004 (9)
N1	0.0171 (10)	0.0220 (12)	0.0309 (13)	−0.0005 (10)	0.0003 (10)	0.0015 (11)
C1	0.0255 (14)	0.0259 (15)	0.0246 (16)	−0.0010 (13)	0.0001 (13)	0.0009 (14)
C2	0.0257 (15)	0.0193 (14)	0.0316 (16)	0.0008 (12)	0.0024 (13)	−0.0037 (13)
C3	0.0163 (13)	0.0167 (13)	0.0316 (16)	−0.0010 (11)	−0.0015 (12)	0.0033 (13)
C4	0.0170 (13)	0.0195 (13)	0.0252 (15)	−0.0042 (11)	−0.0015 (11)	0.0011 (13)
C5	0.0178 (12)	0.0160 (14)	0.0283 (15)	−0.0010 (11)	0.0025 (11)	−0.0022 (13)
C6	0.0188 (13)	0.0188 (14)	0.0255 (15)	−0.0039 (11)	0.0013 (12)	0.0027 (13)
C7	0.0190 (13)	0.0176 (14)	0.0243 (15)	−0.0006 (11)	−0.0014 (11)	0.0003 (12)
C8	0.0180 (12)	0.0246 (15)	0.0274 (15)	−0.0004 (12)	−0.0017 (12)	0.0051 (13)
C9	0.0179 (12)	0.0240 (14)	0.0233 (15)	0.0037 (12)	0.0014 (12)	0.0006 (13)
C10	0.0110 (12)	0.0238 (15)	0.0263 (15)	0.0001 (11)	−0.0047 (11)	−0.0001 (12)
C11	0.0229 (14)	0.0284 (16)	0.0339 (17)	0.0034 (13)	0.0024 (13)	−0.0019 (14)
C12	0.0288 (17)	0.0238 (17)	0.057 (2)	0.0078 (14)	−0.0003 (16)	−0.0010 (16)
C13	0.0293 (15)	0.0245 (17)	0.053 (2)	0.0011 (15)	−0.0081 (16)	0.0161 (16)
C14	0.0293 (16)	0.0375 (19)	0.0315 (17)	−0.0066 (15)	−0.0048 (14)	0.0086 (15)
C15	0.0249 (14)	0.0245 (15)	0.0298 (16)	−0.0007 (13)	−0.0014 (13)	0.0012 (13)
C16	0.0293 (15)	0.0271 (15)	0.0259 (15)	0.0059 (13)	−0.0032 (13)	−0.0018 (14)
C17	0.0235 (14)	0.0289 (17)	0.050 (2)	−0.0086 (13)	0.0014 (15)	0.0005 (16)
C18	0.0220 (14)	0.0352 (17)	0.0389 (18)	0.0019 (13)	0.0051 (14)	0.0042 (16)

### Geometric parameters (Å, °)

**Table:**

O1—C3	1.370 (3)	C9—C10	1.501 (4)
O1—H1O1	0.92 (3)	C9—H9A	0.9800
O2—C4	1.370 (3)	C10—C11	1.382 (4)
O2—C16	1.432 (3)	C10—C15	1.385 (4)
O3—C7	1.435 (3)	C11—C12	1.393 (4)
O3—C9	1.462 (3)	C11—H11A	0.9300
N1—C17	1.455 (4)	C12—C13	1.374 (5)
N1—C7	1.462 (3)	C12—H12A	0.9300
N1—C8	1.468 (3)	C13—C14	1.386 (4)
C1—C6	1.379 (4)	C13—H13A	0.9300
C1—C2	1.394 (4)	C14—C15	1.382 (4)
C1—H1A	0.9300	C14—H14A	0.9300
C2—C3	1.376 (4)	C15—H15A	0.9300
C2—H2A	0.9300	C16—H16A	0.9600
C3—C4	1.397 (4)	C16—H16B	0.9600
C4—C5	1.381 (4)	C16—H16C	0.9600
C5—C6	1.399 (4)	C17—H17A	0.9600
C5—H5A	0.9300	C17—H17C	0.9600
C6—C7	1.505 (4)	C17—H17D	0.9600
C7—H7A	0.9800	C18—H18A	0.9600
C8—C18	1.514 (4)	C18—H18D	0.9600
C8—C9	1.530 (4)	C18—H18B	0.9600
C8—H8A	0.9800		
			
C3—O1—H1O1	108 (2)	O3—C9—H9A	108.7
C4—O2—C16	117.4 (2)	C10—C9—H9A	108.7
C7—O3—C9	108.10 (19)	C8—C9—H9A	108.7
C17—N1—C7	112.8 (2)	C11—C10—C15	118.6 (3)
C17—N1—C8	113.5 (2)	C11—C10—C9	120.3 (3)
C7—N1—C8	102.6 (2)	C15—C10—C9	121.0 (2)
C6—C1—C2	120.8 (3)	C10—C11—C12	120.7 (3)
C6—C1—H1A	119.6	C10—C11—H11A	119.7
C2—C1—H1A	119.6	C12—C11—H11A	119.7
C3—C2—C1	119.7 (3)	C13—C12—C11	119.9 (3)
C3—C2—H2A	120.1	C13—C12—H12A	120.0
C1—C2—H2A	120.1	C11—C12—H12A	120.0
O1—C3—C2	120.0 (3)	C12—C13—C14	120.0 (3)
O1—C3—C4	120.0 (2)	C12—C13—H13A	120.0
C2—C3—C4	119.9 (2)	C14—C13—H13A	120.0
O2—C4—C5	125.7 (2)	C15—C14—C13	119.7 (3)
O2—C4—C3	114.1 (2)	C15—C14—H14A	120.1
C5—C4—C3	120.2 (2)	C13—C14—H14A	120.1
C4—C5—C6	119.9 (3)	C14—C15—C10	121.0 (3)
C4—C5—H5A	120.0	C14—C15—H15A	119.5
C6—C5—H5A	120.0	C10—C15—H15A	119.5
C1—C6—C5	119.4 (2)	O2—C16—H16A	109.5
C1—C6—C7	120.8 (3)	O2—C16—H16B	109.5
C5—C6—C7	119.8 (2)	H16A—C16—H16B	109.5
O3—C7—N1	103.5 (2)	O2—C16—H16C	109.5
O3—C7—C6	111.7 (2)	H16A—C16—H16C	109.5
N1—C7—C6	112.8 (2)	H16B—C16—H16C	109.5
O3—C7—H7A	109.5	N1—C17—H17A	109.5
N1—C7—H7A	109.5	N1—C17—H17C	109.5
C6—C7—H7A	109.5	H17A—C17—H17C	109.5
N1—C8—C18	113.4 (2)	N1—C17—H17D	109.5
N1—C8—C9	101.4 (2)	H17A—C17—H17D	109.5
C18—C8—C9	113.2 (2)	H17C—C17—H17D	109.5
N1—C8—H8A	109.5	C8—C18—H18A	109.5
C18—C8—H8A	109.5	C8—C18—H18D	109.5
C9—C8—H8A	109.5	H18A—C18—H18D	109.5
O3—C9—C10	111.8 (2)	C8—C18—H18B	109.5
O3—C9—C8	104.25 (19)	H18A—C18—H18B	109.5
C10—C9—C8	114.5 (2)	H18D—C18—H18B	109.5
			
C6—C1—C2—C3	−1.3 (4)	C5—C6—C7—N1	−69.3 (3)
C1—C2—C3—O1	−179.5 (2)	C17—N1—C8—C18	73.7 (3)
C1—C2—C3—C4	−0.4 (4)	C7—N1—C8—C18	−164.2 (2)
C16—O2—C4—C5	−9.2 (4)	C17—N1—C8—C9	−164.6 (2)
C16—O2—C4—C3	171.4 (2)	C7—N1—C8—C9	−42.5 (2)
O1—C3—C4—O2	0.7 (3)	C7—O3—C9—C10	−124.5 (2)
C2—C3—C4—O2	−178.4 (2)	C7—O3—C9—C8	−0.3 (3)
O1—C3—C4—C5	−178.7 (2)	N1—C8—C9—O3	26.4 (2)
C2—C3—C4—C5	2.2 (4)	C18—C8—C9—O3	148.2 (2)
O2—C4—C5—C6	178.4 (2)	N1—C8—C9—C10	148.9 (2)
C3—C4—C5—C6	−2.3 (4)	C18—C8—C9—C10	−89.3 (3)
C2—C1—C6—C5	1.2 (4)	O3—C9—C10—C11	−136.8 (2)
C2—C1—C6—C7	−174.8 (2)	C8—C9—C10—C11	104.9 (3)
C4—C5—C6—C1	0.6 (4)	O3—C9—C10—C15	45.9 (3)
C4—C5—C6—C7	176.7 (2)	C8—C9—C10—C15	−72.4 (3)
C9—O3—C7—N1	−26.2 (2)	C15—C10—C11—C12	0.7 (4)
C9—O3—C7—C6	−147.9 (2)	C9—C10—C11—C12	−176.7 (3)
C17—N1—C7—O3	165.9 (2)	C10—C11—C12—C13	0.5 (4)
C8—N1—C7—O3	43.3 (2)	C11—C12—C13—C14	−1.0 (5)
C17—N1—C7—C6	−73.2 (3)	C12—C13—C14—C15	0.3 (5)
C8—N1—C7—C6	164.3 (2)	C13—C14—C15—C10	0.9 (4)
C1—C6—C7—O3	−137.2 (3)	C11—C10—C15—C14	−1.4 (4)
C5—C6—C7—O3	46.8 (3)	C9—C10—C15—C14	176.0 (3)
C1—C6—C7—N1	106.7 (3)		

### Hydrogen-bond geometry (Å, °)

**Table:**

Cg1 is the centroid of the C10–C15 phenyl ring.

**Table:**

*D*—H···*A*	*D*—H	H···*A*	*D*···*A*	*D*—H···*A*
O1—H1O1···O3^i^	0.92 (4)	2.08 (3)	2.909 (3)	148 (3)
C5—H5A···O1^ii^	0.93	2.42	3.244 (3)	148
C16—H16A···Cg1^iii^	0.96	2.91	3.628 (3)	133

Symmetry codes: (i) −*x*+1, *y*−1/2, −*z*+1/2; (ii) −*x*+1, *y*+1/2, −*z*+1/2; (iii) −*x*+3/2, −*y*−1, *z*+1/2.
